# Poly(D,L-Lactic Acid) Nanoparticle Size Reduction Increases Its Immunotoxicity

**DOI:** 10.3389/fbioe.2019.00137

**Published:** 2019-06-06

**Authors:** Jessica Da Silva, Sandra Jesus, Natália Bernardi, Mariana Colaço, Olga Borges

**Affiliations:** ^1^Center for Neuroscience and Cell Biology, University of Coimbra, Coimbra, Portugal; ^2^Faculty of Pharmacy, University of Coimbra, Azinhaga de Santa Comba, Coimbra, Portugal

**Keywords:** polylactic acid, poly(D,L-lactic acid), polymeric nanoparticles, drug delivery systems, immunotoxicity, size-dependent cytotoxicity, hemocompatibility, cell culture medium

## Abstract

Polylactic acid (PLA), a biodegradable and biocompatible polymer produced from renewable resources, has been widely used as a nanoparticulate platform for antigen and drug delivery. Despite generally regarded as safe, its immunotoxicological profile, when used as a polymeric nanoparticle (NP), is not well-documented. Thus, this study intends to address this gap, by evaluating the toxicity of two different sized PLA NPs (PLA_A_ NPs and PLA_B_ NPs), produced by two nanoprecipitation methods and extensively characterized regarding their physicochemical properties in *in vitro* experimental conditions. After production, PLA_A_ NPs mean diameter (187.9 ± 36.9 nm) was superior to PLA_B_ NPs (109.1 ± 10.4 nm). Interestingly, when in RPMI medium, both presented similar mean size (around 100 nm) and neutral zeta potential, possibly explaining the similarity between their cytotoxicity profile in PBMCs. On the other hand, in DMEM medium, PLA_A_ NPs presented smaller mean diameter (75.3 ± 9.8 nm) when compared to PLA_B_ NPs (161.9 ± 8.2 nm), which may explain its higher toxicity in RAW 264.7. Likewise, PLA_A_ NPs induced a higher dose-dependent ROS production. Irrespective of size differences, none of the PLA NPs presented an inflammatory potential (NO production) or a hemolytic activity in human blood. The results herein presented suggest the hypothesis, to be tested in the future, that PLA NPs presenting a smaller sized population possess increased cytotoxicity. Furthermore, this study emphasizes the importance of interpreting results based on adequate physicochemical characterization of nanoformulations in biological medium. As observed, small differences in size triggered by the dispersion in cell culture medium can have repercussions on toxicity, and if not correctly evaluated can lead to misinterpretations, and subsequent ambiguous conclusions.

## Introduction

Polylactic acid (PLA) is a Food and Drug Administration approved polymer that has proven to be a very versatile material, with interesting properties such as biocompatibility and biodegradability (Essa et al., 2012; Legaz et al., 2016). Thus, PLA has been explored regarding many therapeutic applications, including as a nanoparticulate antigen and drug delivery vehicle (Essa et al., 2012; Legaz et al., 2016).

The great interest in using nanoparticles (NPs) for biomedical applications (Jiao et al., 2014) is transversal to various polymeric materials, despite the poorly understood correlation between their physicochemical properties and their effects on the immune system. This knowledge gap partially results from the fact that NPs physicochemical properties, particularly its reduced size, hinder the application of traditional toxicity assays and further contribute to the misinterpretation of results and ambiguous conclusions among research groups (Dobrovolskaia et al., 2009). Additionally, the mandatory use of biological medium during toxicological assays can modify the NPs characteristics such as size, surface charge and morphology, through phenomenon's like protein corona formation and particle agglomeration, which will therefore influence the immunotoxicity profile of the NPs (Kendall et al., 2014). Therefore, a detailed characterization of the NPs in the experimental assay conditions is crucial to discuss the results, but is commonly absent from the scientific published reports. Biodegradable polymers such as PLA are generally regarded as safe, but their immunotoxicological profile when used as NPs, is not well documented (Singh and Ramarao, 2013). Previously, da Luz et al. has published an interesting paper assessing the toxicity and biocompatibility of PLA NPs in A549 cells (da Luz et al., 2017). Similarly, Legaz et al. conducted toxicity studies in Schneider's D. melanogaster line 2 (S2) cells (Legaz et al., 2016).

In this study, we described the production method of two different sized PLA NPs (PLA_A_ NPs and PLA_B_ NPs), in order to evaluate how the NP size affects their toxicological profile using cells from the immune system. *In vitro* immunotoxicity studies comprised hemocompatibility assays, cell viability experiments with peripheral blood mononuclear cells (PBMCs) and RAW 264.7 macrophage cell line, and nitric oxide (NO) and reactive oxygen species (ROS) production assays in RAW 264.7 cells. Furthermore, for the discussion of these results we have included the characterization of both PLA NPs regarding its size, polydispersity index (PDI) and zeta potential in the different cell culture media used in *in vitro* studies. In contrast to other published reports evaluating the toxicity of PLA NPs, this report aims to highlight the importance of the NPs characterization under *in vitro* experimental conditions for the establishment of relationships between the NPs properties and their effect in cells of the immune system. Not being an exhaustive study of immunotoxicology, it nevertheless intends to emphasize the importance of these studies in the development of nanomedicines.

## Materials and Methods

### Poly(D,L-Lactide) Polymer

Poly(D,L-lactide) (PDLLA) polymer with an average molecular weight (MW) of 1,01,782 g/mol [analyzed by gel permeation chromatography/size exclusion chromatography (GPC/SEC)] and an inherent viscosity of 0.68 dL/g was obtained from Sigma-Aldrich Corporation (St. Louis, MO, USA).

### PLA NP Production

For PLA_A_ NPs production, PDLLA was dissolved at 2 mg/mL in acetone. NPs formed spontaneously upon dropwise addition of 4.5 mL of PDLLA solution to 13.5 mL of an aqueous solution (pyrogen-free water) with 1% of Pluronic® F68 Prill (Basf Corporation, Ludwigshafen, Germany) using a high-speed homogenizer at 13,000 rpm. The homogenization was maintained for another 2 min, after total addition of the PDLLA solution. The PLA_A_ NPs were concentrated by centrifugation at 13,000 g for 20 min at 10°C, resuspended in pyrogen-free water and concentrated again. This procedure was repeated 2 more times, and finally each batch was concentrated in a final volume of 2 mL. On the other hand, for the production of PLA_B_ NPs, PDLLA was dissolved at 0.75 mg/mL in acetone. NPs formed spontaneously upon dropwise addition of 1 mL of PDLLA solution to 2.5 mL of an aqueous solution with 0.1% of Pluronic F68 using a vortex homogenizer and the agitation was maintained for another 2 min, after the total addition of the PDLLA solution. In order to concentrate and wash the NPs, 8 batches of PLA_B_ NPs (20 mL) were centrifuged with Vivaspin 20 centrifugal concentrator (MWCO 300 KD, ThermoFisher Scientific Inc., Waltham, MA, USA) at 3,000 g at 10°C until <1 mL was recovered in the centrifuge tube. The NPs were then resuspended in 10 mL pyrogen-free water, the centrifugation procedure was repeated, and the NPs were resuspended in a final volume of 1 mL pyrogen-free water. *In vitro* experiments and the respective characterization in *in vitro* conditions, were performed by diluting these concentrated NP suspensions in serum supplemented cell culture media as described below.

### PLA NP Characterization

Zetasizer Nano ZS (Malvern Instruments, Ltd., Worcestershire, UK) was used to measure particle size, and the respective polydispersity index (PDI), by dynamic light scattering (DLS) and particle zeta potential through electrophoretic light scattering (ELS). The samples were characterized dispersed in pyrogen-free water and in supplemented culture media (RPMI and DMEM). In the second case, the size and zeta potential assessment was done immediately after dilution in the culture medium, and after 24 h of incubation at 37°C. The NP size when dispersed in pyrogen-free water was also confirmed by transmission electron microscopy (TEM). Samples were placed on a microscopy grid and observed under a FEI-Tecnai G2 Spirit Biotwin, a 20–120 kV TEM (FEI Company, OR, USA).

### Immunotoxicity and Hemocompatibility Assays

#### *In vitro* Studies With Human Blood

##### Hemolysis assay

Hemolysis assay was performed according to published protocols with minor modifications (Pattani et al., 2009; Villiers et al., 2009). Whole blood was collected from healthy donors after formal acceptance with a written informed consent. Blood was diluted with PBS to adjust total blood hemoglobin (TBH) concentration to 10 ± 2 mg/mL (TBHd). A volume of 100 μL of PLA NPs suspensions, PBS (negative control), or Triton-X-100 (positive control) were added to 700 μL PBS in different tubes. Then, 100 μL of TBHd was added to each tube, followed by incubation at 37°C for 3 h ± 15 min. NPs were also incubated with PBS without blood to evaluate the possible NP interference. After the incubation time, the tubes were centrifuged at 800 g for 15 min. One hundred microliter of each supernatant and 100 μL cyanmethemoglobin (CMH) reagent were added to a 96-well-plate. The CMH reagent was prepared by mixing 1000 mL Drabkin's reagent and 0.5 mL of 30% Brij 35 solution (Sigma-Aldrich, St. Louis, MO, USA). The absorbance (OD) at 540 nm was determined and the percentage of hemolysis was calculated by Equation 1:

(1) $$\begin{array}{r} Hemolysis\;\left(\%\right)=\;\frac{\left(OD\;sample\;\left(540\;nm\right)-OD\;PBS\;\left(540\;nm\right)\right)}{\left(OD\;TBHd\;\left(540\;nm\right)-OD\;PBS\;\left(540\;nm\right)\right)}x\;100 \end{array}$$

#### *In vitro* Studies With PBMCs

##### PBMCs isolation

Buffy coats obtained from normal donors (heparinized syringes) were kindly given by IPST, IP (Coimbra, PT). PMBCs were isolated on a density gradient with Lymphoprep (Axis-Shield, Dundee, Scotland) according to the provider's guidance protocol and as published by our group (Jesus et al., 2017). Isolated PBMCs were cultured in Roswell Park Memorial Institute medium (RPMI) with 10% heat inactivated fetal bovine serum (FBS), supplemented with 2 mM L-glutamine, 1% penicillin/streptomycin and 20 mM HEPES.

##### Nanoparticle toxicity

PLA NPs cytotoxicity was evaluated on human PBMCs using 3-(4,5-dimethylthiazol-2-yl)-2,5-diphenyltetrazolium bromide (MTT) assay. Cells were plated in a 96-well plate at a density of 5 x 10^5^ monocytes/well. Serial dilutions of NPs and controls were incubated with the cells for 24 h, at 37°C and 5% CO_2_. After this period, 20 μL of MTT solution (5 mg/mL) in PBS were added to each well-followed by additional 4 h incubation. To ensure dissolution of the formazan crystals, cell culture plates were centrifuged (800 g, 25 min, 20°C) and the culture medium was replaced by DMSO and the OD of the resultant colored solution was measured at 540 and 630 nm. Cell viability (%) was calculated by Equation 2:

(2) $$\begin{array}{r} Cell\;viability\;\left(\%\right)\\ \;=\frac{\left(OD\;sample\;\left(540\;nm\right)-OD\;sample\;\left(630\;nm\right)\right)}{\left(OD\;control\;\left(540\;nm\right)\;-\;OD\;control\;\left(630\;nm\right)\right)}\times 100 \end{array}$$

The inhibitory concentration for 50% of cell viability (IC_50_) was calculated by plotting the log concentration of the NPs vs. inhibition percentage of cell viability and extrapolating the value from a non-linear regression using Prism 6.0 (GraphPad Software, San Diego, CA, USA).

Cytotoxicity results obtained with MTT assay were confirmed with propidium iodide (PI) assay. Briefly, cells incubated with 4 nanoparticle concentrations previously used in MTT assay were centrifuged (800 g, 25 min, 20°C), resuspended in PBS and collected for analysis in a BD FACSCalibur Flow Cytometer (BD Biosciences, Bedford, MA, USA) using PI solution (0.5 μg/mL).

#### *In vitro* Studies With RAW 264.7 Macrophage Cell Line

RAW 264.7 (ATCC® TIB-71™) were acquired to ATCC (Manassas, VA, USA), cultured in Dulbecco's Modified Eagle Medium (DMEM) supplemented with 10% heat inactivated FBS, 1% Penicillin/Streptomycin, 10 mM HEPES and 3.7 g/L Sodium Bicarbonate, and used until passage 18.

##### Nanoparticle cytotoxicity

PLA NP toxicity in RAW 264.7 was assessed as described previously for PBMCs with some modifications. Briefly, for MTT assay, macrophages were plated at a concentration of 2 × 10^4^ cells/well and the incubation with MTT solution was performed for 1 h 30 min.

For the assay with PI, the cells were collected using the dissociation medium (PBS-EDTA 5 mM) followed by centrifugation (250 g, 10 min, 20°C) to replace the medium with PBS.

##### Nanoparticle effect on production of the reactive oxygen species

The ROS production was assessed using the dichlorofluorescein diacetate probe (DCFH-DA) (Thermo Fisher Scientific Inc., Waltham, MA, USA). The RAW 264.7 cells were incubated in a black 96-well plate for 24 h at 37°C and 5% CO_2_, at density of 0.5 × 10^5^ cells/well. After that period, serial dilutions of PLA NPs were incubated with the cells, to evaluate ROS stimulation. LPS was used as a positive control (1 μg/mL).

After 24 h, cell culture medium was replaced by DCFH-DA (50 μM) in serum free DMEM and the cells were incubated for another 2 h at 37°C and 5% CO_2_. The resulting fluorescence was read at 485/20 nm and 528/20 nm (excitation/emission wavelengths).

To calculate the stimulation of ROS production, Equation (3) was applied:

(3) $$\begin{array}{r} ROS\;production\;\left(mean\;fluorescence\;increase\right)\\ =\;\frac{Fluorescence_{SAMPLE}}{Fluorescence_{NEGATIVE\;CONTROL}} \end{array}$$

##### Nanoparticle effect on nitric oxide production

The NO production by RAW 264.7 was evaluated based on nitrite quantification using the Griess reagent. RAW 264.7 cells were incubated in a 48-well-plate at a density of 2.25 × 10^5^ cells/well for 24 h at 37°C and 5% CO_2._ After that period, cell culture medium was replaced by serial dilutions of PLA NPs diluted in cell culture medium without phenol red. LPS was used as a positive control (1 μg/mL). To test if the NPs were able to inhibit LPS stimulated NO production, the same NP concentrations were incubated together with cells in the presence of the LPS (1 μg/mL).

The Cell supernatants were collected 24 h after incubation, and 100 μL of each test sample was plated in a 96-well-plate and combined with 100 μL of Griess reagent. A calibration curve performed with sodium nitrite (0–80 μg/mL) was also plated in duplicate. The optical density of the samples was measured at 550 nm and NO quantification was extrapolated from the calibration curve.

To calculate the inhibition of NO production upon stimulation with LPS (Equation 4) was applied:

(4) $$\begin{array}{r} Inhibition\;of\;NO\;production\;\left(\%\right)\\ \;=\;\frac{{NO\left(\text{μ}g/mL\right)\;}_{SAMPLE}}{{NO\;\left(\text{μ}g/mL\right)\;}_{POSITIVE\;CONTROL}}\times 100 \end{array}$$

### Statistical Analysis

Data were analyzed using GraphPad Prim 6 (GraphPad Software, Inc., La Jolla, CA, USA), in which significant differences were obtained from one-way ANOVA, and values were considered statistically different when *p* < 0.05. *In vitro* data were expressed as means ± standard error of the mean (SEM).

## Results

### PLA_A_ NPs Are the Largest in Water but the Smallest in Culture Medium

**Figure 1:**
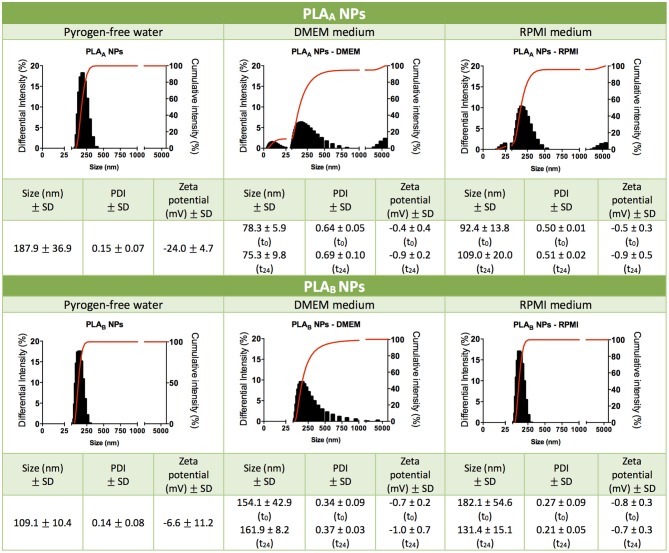
Characterization of PLA NPs. Particle mean size distribution (nm), polydispersity index (PDI), zeta potential (mV) and illustrative graphics of differential and cumulative intensities, after concentration and resuspension in pyrogen-free water, or after 24 h of incubation in cell culture media (DMEM medium or RPMI medium). Data are presented as mean ± standard deviation (SD), *n* ≥ 3 (three or more independent experiments, each in triplicate).

**Figure 2:**
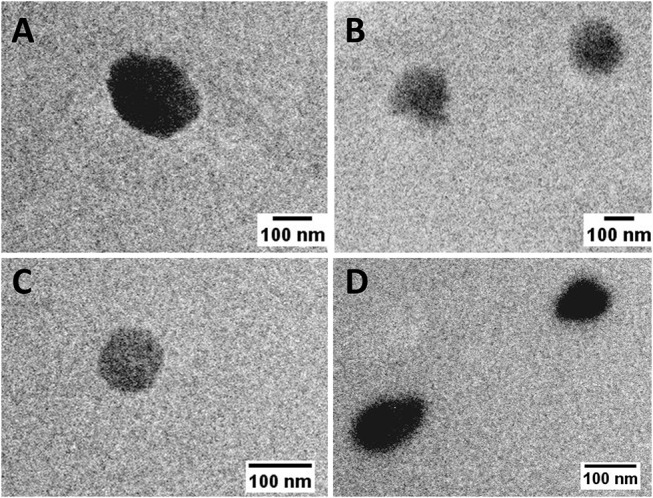
TEM images of PLA NPs dispersed in pyrogen-free water (scale bar: 100 nm). **(A,B)** PLA_A_ NPs; **(C,D)** PLA_B_ NPs.

Although PLA polymer has been approved by FDA for human use in an extensive range of applications (Tyler et al., 2016). The information about its toxicological profile when used as a NP is scarce (Singh and Ramarao, 2013). In order to give new insights on the relationship between NP physicochemical properties and their immunotoxicity, two different sized PLA NPs were produced and characterized regarding their mean size, PDI and zeta potential (Figure 1). PLA_A_ NPs presented a mean diameter of 187.9 ± 36.9 nm and a zeta potential of −24.0 ± 4.7 mV in pyrogen-free water, while PLA_B_ NPs presented a mean diameter of 109.1 ± 10.4 nm and a zeta potential of −6.6 ± 11.2 mV, both presenting a low PDI compatible with only one narrow-size population of particles (see graphics on Figure 1). The more negative charge of PLA_A_ NPs could be explained by the higher concentration of Pluronic F68 used in the NP production method, since increased surface layer of surfactant may decreases the NPs zeta potential (Santander-Ortega et al., 2006). Sizes were also analyzed after dispersion in cell culture media, in order to evaluate the stability of the NPs in the experimental assay conditions. These tests were performed right after dispersion in DMEM and RPMI, and 24 h after incubation at 37°C. Results from initial dispersion and after 24 h incubation were comparable (Figure 1), so the 24 h-incubation period did not altered the characteristics of the particles. However, great differences, when compared with the initial size (pyrogen-free water), were observed when the particles were suspended in RPMI, but especially in DMEM. In case of PLA_A_ NPs, the size decreased and in case of PLA_B_ NPs the size increase. To better understand the differences, representative graphs of differential and cumulative intensities of size distribution were obtained for both particles, suspended in pyrogen-free water and after 24 h incubation in RPMI and DMEM. When comparing the PLA_A_ graphs from cell culture media with the ones obtained in the original medium (pyrogen-free water), we observed the appearance of 3 size-populations, compatible with a higher PDI. To highlight, the appearance of a small size population of particles explaining the decrease of the mean size diameter. The same phenomenon was not observed with PLA_B_ NPs. On the contrary, in RPMI the size remained unaltered and in DMEM the size increased as a result of some aggregation of the particles. In order to confirm the initial differences in size between PLA_A_ NPs and PLA_B_ NPs, TEM images were obtained with particles dispersed in pyrogen free water (Figure 2). As illustrated, both NPs are round shaped and sizes confirmed the DLS measurements.

### Both PLA NPs Present a Good Hemocompatibility Profile

Hemolysis is the breakdown of red blood cells with subsequent release of intracellular contents. *In vivo*, this can lead to anemia or other pathological conditions (Dobrovolskaia et al., 2008). It is important to assess the NP effect on these blood elements not only when the intravenous route of administration is considered but also when addressing other administration routes, in order to establish their hemocompatibility (Dobrovolskaia et al., 2008). For that reason, PLA NP hemocompatibility was assessed in human whole blood and hemolytic values were considered above 5%, as recommended by American Society for Testing and Materials International (ASTM, 2013).

**Figure 3:**
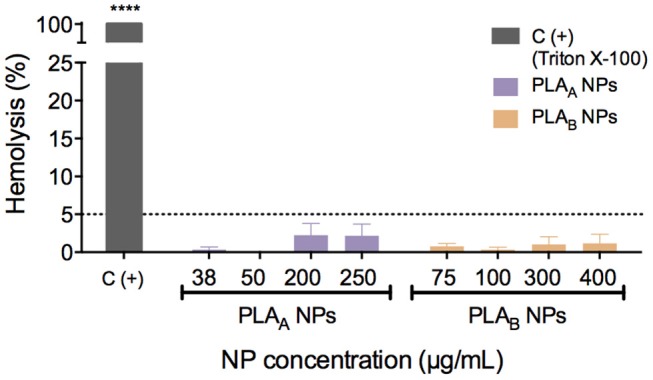
Hemolytic activity of PLA NPs in human blood after 3 h of incubation at 37°C. Triton X-100 was used as a positive control. Hemolytic values were considered above 5%, as recommended by American Society for Testing and Materials International (ASTM, 2013). Data are presented as mean ± SEM, *n* ≥ 3 (three or more independent experiments, each in duplicate). ****p < 0.0001 indicates values that differ significantly from negative control (PBS).

The results from Figure 3 showed that both PLA NPs (A and B) have a good hemocompatibility profile, since none induced hemolysis above 5%, considering the concentration range tested (38–250 μg/mL for PLA_A_ NPs and 75–400 μg/mL for PLA_B_ NPs).

### PLA_A_ NPs Show a Pronounced Cytotoxicity Profile in Comparison to PLA_B_ NPs in RAW 264.7

The colorimetric MTT assay for measuring cell metabolic activity is based on the cellular conversion of a tetrazolium salt (MTT) into an insoluble formazan, that can be dissolved in DMSO generating a purple signal (Altmeyer et al., 2016). Therefore, through an indirect way, MTT assay was used to evaluate the cytotoxicity of PLA NPs after 24 h incubation with PBMCs and RAW 264.7.

**Figure 4:**
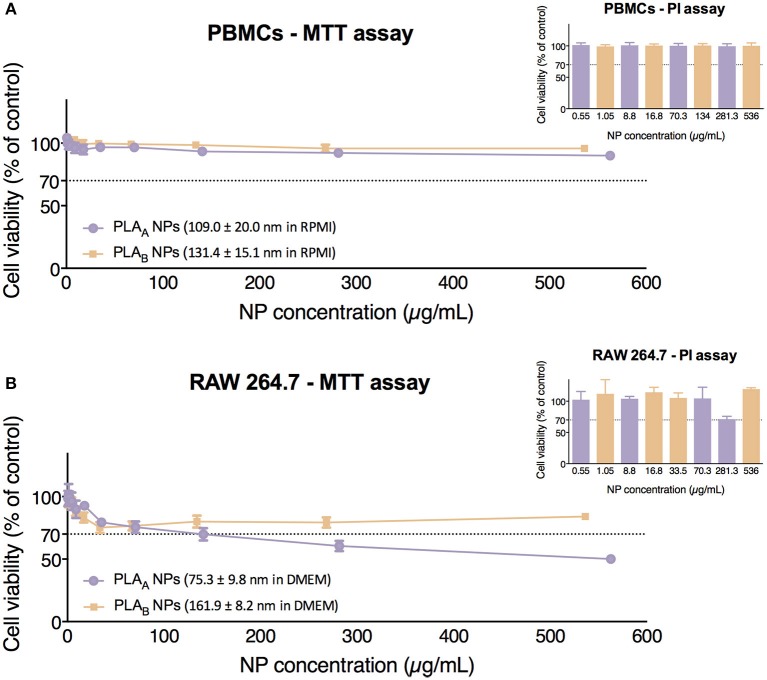
Cytotoxicity assays (MTT and PI) performed in PBMCs **(A)** and in RAW 264.7 cell line **(B)** after 24 h of incubation with PLA NPs. Data are presented as mean ± SEM, *n* ≥ 4 (four or more independent experiments, each in triplicate).

Results presented in Figure 4A show that neither PLA_A_ NPs nor PLA_B_ NPs induced cytotoxicity in PBMCs, since the incubation with both resulted in cell viabilities above 70% under the concentration range tested (0.55–562.5 μg/mL for PLA_A_ NPs and 1.05–536 μg/mL for PLA_B_ NPs). Importantly, the similarity in the cytotoxicity profile in this primary culture could be explained by the similar mean diameter and zeta potential of PLA NPs when dispersed in RPMI medium. In fact, the differences in size previously seen in water were masked in RPMI.

Concerning cytotoxicity in RAW 264.7, we can observe in Figure 4B that PLA_A_ NPs presented a higher cytotoxicity than PLA_B_ NPs, since they presented an estimated IC_50_ of 540.6 μg/mL, while with PLA_B_ NPs cell viabilities below 70% were never observed, and therefore the estimation of IC_50_ was not possible under the concentration range tested (1.05–536 μg/mL). These results are probably correlated with the size of the both PLA NPs in DMEM (RAW 264.7) medium. In case of the PLA_A_ NPs, the presence of great NP population with a size below 25 nm might explain their higher cytotoxicity.

In these experiments, the control with the stock solution vehicle (mainly pyrogen-free water from the last NP wash), was tested in the volume correspondent to the highest NP concentration and no decrease in cell viability was verified. These controls ensured that the decrease in cell viability is from the NPs in suspension and not the vehicle of the NP suspension.

In order to avoid possible excessive assumption regarding cytotoxicity when using only a metabolic assay, these results were confirmed with PI assay, which evaluates the integrity of the cell membrane. Results for PLA_A_ NPs and PLA_B_ NPs in both cellular models were similar to the ones obtained with MTT (Figure 4) and confirmed the higher toxicity of PLA_A_ NPs in RAW 264.7 cells.

### PLA_A_ NPs but Not PLA_B_ NPs Induce a Significant Concentration-Dependent ROS Production

**Figure 5:**
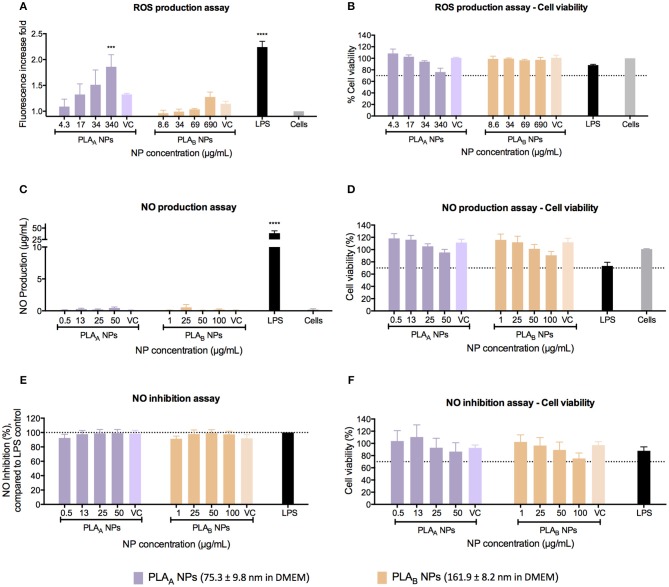
Immunotoxicity assays, performed in RAW 264.7 macrophage cell line after 24 h of incubation with PLA NPs and PLA NPs dispersion media (VC). **(A)** ROS production assay using LPS as a positive control and unstimulated cells as a negative control. Results are presented in fluorescence increase fold compared to the negative control. **(B)** Cell viability assay (MTT) after the performance of ROS production assay. **(C)** NO production assay using LPS as a positive control and unstimulated cells as a negative control. **(D)** Cell viability assay (MTT) after the performance of NO production assay. **(E)** NO inhibition assay. For the estimation of PLA NPs inhibitory effect on NO production, PLA NPs were incubated simultaneously with LPS. The percentage of NO inhibition was calculated considering 100% the NO production induced by LPS without PLA NPs **(F)**. Cell viability assay (MTT) after the performance of NO inhibition assay. Data are presented as mean ± SEM, *n* ≥ 3 (three or more independent experiments, each in triplicate). ***p < 0.001 and ****p < 0.0001.

The ROS, such as superoxide or hydrogen peroxide, are continually produced during metabolic processes (Brüne et al., 2013; Kwon et al., 2017). ROS generation is normally counterbalanced by the action of antioxidant enzymes and other redox molecules (Brüne et al., 2013; Kwon et al., 2017). However, when overproduced by activated macrophages, ROS can lead to cellular injury (Circu and Aw, 2010; Brüne et al., 2013; Kwon et al., 2017). It has been proven by Saini and co-workers that NPs may promote apoptotic cell death, through the induction of oxidative stress by accumulating ROS (Saini et al., 2016). Therefore, it is important to evaluate the potential effect of PLA NPs in ROS production. This assay was performed using the cell-permeable fluorogenic probe DCFH-DA, which can be detected on a standard fluorometric plate reader (Zolnik et al., 2011). ROS production assay in RAW 264.7 was performed after 24 h of incubation and as demonstrated in Figure 5A, there was a concentration-dependent ROS production for PLA_A_ NPs. The same effect was not observed for the PLA_B_ NPs, even considering that a lower PLA_A_ NP concentrations were tested, when compared with PLA_B_ NPs (4.3–340 μg/mL for PLA_A_ NPs and 8.6–690 μg/mL for PLA_B_ NPs). We could hypothesize that this concentration-dependent ROS production is an indication of cellular toxicity, as demonstrated by the cell viability assay in the Figure 5B, where for the higher PLA_A_ NP concentration the resultant cellular viability was near 70%. For PLA_B_ NPs it was observed an increased trend of ROS production. However, the values observed were not statistically different from the unstimulated cells. Furthermore, in opposition to the results of PLA_A_ NPs, no trend for decrease in cell viability was shown for PLA_B_ NPs (Figure 5B).

### PLA NPs Do Not Have an Inflammatory Potential in RAW 264.7

The NO is a reactive nitrogen specie, produced by nitric oxide synthase enzymes (Boscá et al., 2005; Caruso et al., 2017). It is an important inflammatory mediator released by macrophages during inflammation, and is one of the main cytostatic, cytotoxic, and pro-apoptotic mechanisms of the immune response (Boscá et al., 2005; Caruso et al., 2017). In order to assess the inflammatory or anti-inflammatory properties of PLA NPs, NO production by RAW 264.7 cells was measured using the Griess reaction method after 24 h of incubation with different test samples.

The pro-inflammatory effect of PLA NPs was evaluated by measuring the NO release upon stimulation with NPs, and the anti-inflammatory effect was evaluated by measuring the ability of the NPs to inhibit NO release induced by LPS. In the first approach, none of the PLA NPs induced NO production under the concentration range tested (0.5–50 μg/mL for PLA_A_ NPs and 1–100 μg/mL for PLA_B_ NPs) (Figure 5C). Importantly, these concentrations were chosen because they did not induce significant cellular death under the assay conditions, and higher concentrations would result in cellular death above 30%, which could compromise NO production (Figure 5D).

The second approach, using the same concentration ranges, revealed that both PLA NPs did not inhibited the NO production stimulated with LPS (Figure 5E) and test conditions did not significantly reduce cell viability (Figure 5F).

## Discussion

According to our results, PLA NPs did not present hemolytic activity in concentrations up to 250 and 400 μg/mL for PLA_A_ and PLA_B_ NPs, respectively. Importantly, these are very high concentrations, far from the reality of *in vivo* administrations. In fact, apart from the fact that the experiment is performed with diluted blood (>10 times diluted), 250 μg/mL would correspond to a intravenously injected human dose of 1400 mg of NP and 400 μg/mL to a dose of 2240 mg [in a 70 kg person, with 5.6 L of blood (Dobrovolskaia and McNeil, 2013)]. Results confirm therefore the hemocompatibility of PLA NPs and are accordant with Altmeyer and co-workers, who described that no erythrocyte damage is caused by blank PLA NPs produced by an emulsion/solvent evaporation method with polyvinyl alcohol (PVA) (Altmeyer et al., 2016).

One of the most important conclusions herein presented is that even small changes in the physicochemical characteristics of similar NPs can originate different cytotoxicity profiles. In detail, results from RAW 264.7 suggested that PLA_A_ NPs induced the higher toxicity, and data from the NP characterization in the experiment conditions revealed these NPs presented the smaller mean diameter, resultant from a higher heterogeneity of the NP population, with emphasize for a population presenting a mean diameter of 10 nm. However, in PBMCs, both PLA NPs presented a similar cytotoxicity profile. Interestingly, in RPMI medium, used for PBMCs experiments, PLA_A_ NPs and PLA_B_ NPs presented a similar mean size and a more similar size-distribution profile than in DMEM medium. Considering these results, we can hypothesize that the smaller NP population in PLA_A_ NPs, resultant from a modification after dispersion in cell culture medium, is contributing to the increased toxicity of PLA_A_ NPs. These results are concordant with the concept that smaller NP can induce more cellular damages, due to increased ability to enter the cells, and particularly, sizes <10 nm can even reach the cell nucleus (Sukhanova et al., 2018). For instance, in a recent study (da Luz et al., 2017) it was proposed that small sized PLA NPs were mainly internalized in A549 cells through clathrin-coated pits in detriment of other endocytic pathways. In the future, the assessment of the mechanisms involved in the uptake of PLA_A_ NPs and PLA_B_ NPs could help clarify the cause of increased toxicity observed in PLA_A_ NPs.

The evaluation of the ROS production confirmed the correlation of the different toxicity profile of PLA NPs with the NP physicochemical differences and highlighted the importance of performing case-by-case evaluations. In fact, we demonstrated that PLA_A_ NPs induced a concentration-dependent ROS production, whereas PLA_B_ NPs did not stimulate statistically significant ROS production even with higher concentrations. A published report from Singh and co-workers (Singh and Ramarao, 2013) suggested that PLA NPs (emulsion-diffusion-evaporation method using PVA) induced no effect on ROS production up to 100 μg/mL concentration, whereas 300 μg/mL showed 1.5- to 2-fold stimulation of ROS production. Their results are in agreement with ours for PLA_A_ NPs, however, they are not aligned with the results from PLA_B_ NPs. This stresses the importance of an adequate evaluation when testing distinct polymeric nanomaterials rather than excessively extrapolating conclusions.

According to literature, PLA may induce inflammatory responses, due to its hydrophobicity, lack of bioactivity, and release of acidic degradation by-products (Li and Chang, 2004; Farah et al., 2016; Yoon et al., 2017). Nevertheless, this study showed that PLA polymer properties are not fully exchangeable with nanosized PLA particles. Actually, we showed that both PLA NPs produced within this study did not present effects on NO production under the concentration range tested, suggesting it does not induce an inflammatory response in RAW 264.7.

Importantly, during the execution of these studies we also hypothesized that the accentuated toxicity profile presented by PLA_A_ NPs could be related to the use of a higher concentration of Pluronic F68 during the production. Despite we have washed the PLA_A_ NPs more exhaustively than PLA_B_ NPs to remove the surfactant, the negative zeta potential in pyrogen-free water gave an indication that PLA_A_ NPs could have more surfactant on its surface. To better understand whether PLA_A_ NPs accentuated effect on ROS production could result from Pluronic F68, the assay was repeated using a range of surfactant dilutions in water (0.00025–0.25%) and no pro-oxidative effect was verified and also no decrease in cell viability.

Lastly, polymeric NPs application into clinical research is dependent on more accurate knowledge of the NP interactions with the human body (Hoshyar et al., 2016). To address this issue, well-executed *in vitro* studies are needed to establish relationships between their biological activity and their physicochemical properties, such as the NP size (Hoshyar et al., 2016). In this sense, exploiting PLA NPs properties correlation with toxicity in a rigorous way represented an interesting challenge for our research group. Accordingly, important recommendations were considered for the development of this work, such as the detailed characterization of the NP physicochemical properties in the original medium (pyrogen-free water) and in *in vitro* assay conditions, the inclusion of positive and negative controls, as well as the assessment of the NP interference before implementing testing protocols. To highlight, in every experiment, the NPs solvent (vehicle control) were also evaluated, in order to ensure that the observed effects were specific from PLA NPs. Also, for cytotoxicity assessment, more than one cell type was used to estimate the same endpoint, and two different methodologies (MTT and PI) were employed to confirm the results. These details shall increase the results reliability and relevance, as extensively discussed by (Drasler et al., 2017).

## Conclusion

In this study, we observed that size highly influences PLA NPs toxicity profile. A new hypothesis to be confirmed in future arose in the course of this work. The smaller NPs are able to induce higher cellular toxicity, particularly mediated by ROS production. Nevertheless, the effect of size was only accurately addressed after characterization in *in vitro* assay conditions. Indeed, we exposed the influence of cell culture media on these polymeric NPs physicochemical characteristics and the respective repercussions on their toxicity. This report illustrates how an adequate NP characterization is crucial, in order to avoid misinterpretations, and consequent ambiguous conclusions. This remark can be further transposable to *in vivo* conditions, since the contact of NP with biological solutions, such as blood, saliva, nasal or gastric fluids can change the NP physicochemical properties, and those are known to be essential for the generation of a biological effect (Oh and Park, 2014).

We strongly believe that this study will help other research groups to achieve better understanding of their results and to obtain improved conclusions supporting the current scientific evidence.

## Data Availability

All datasets generated for this study are included in the manuscript and/or the supplementary files.

## Author Contributions

JD carried out the experimental work. JD, SJ, and OB conceived and planned the experiments. All authors discussed the results and contributed to the final manuscript, reading, and approving the submitted version.

### Conflict of Interest Statement

The authors declare that the research was conducted in the absence of any commercial or financial relationships that could be construed as a potential conflict of interest.
